# Host–Microbiome Interactions in Chronic Itch

**DOI:** 10.3390/jcm14165633

**Published:** 2025-08-09

**Authors:** Tammy Gonzalez, Sophie M. Bilik, Olivia M. Burke, Irena Pastar, Gil Yosipovitch

**Affiliations:** Dr. Phillip Frost Department of Dermatology and Cutaneous Surgery, University of Miami Miller School of Medicine, Miami, FL 33136, USA

**Keywords:** chronic itch, skin microbiome, microbial dysbiosis, neuroimmune signaling, prurigo nodularis, lichen simplex chronicus, antimicrobial peptides, protease-activated receptors, IL-31, TRP channels

## Abstract

Chronic itch is a debilitating condition characterized by persistent pruritus lasting more than six weeks, significantly impairing quality of life. While the role of the immune system and neural circuits in itch is increasingly understood, the contribution of the skin microbiome, especially in non-atopic itch disorders, remains underexplored. This review synthesizes emerging evidence on how microbial dysbiosis contributes to chronic pruritus through multiple molecular pathways: disruption of skin barrier integrity, modulation of neuroimmune signaling axes, and direct activation of pruriceptors. We highlight recent studies identifying microbiome shifts in prurigo nodularis (PN) and lichen simplex chronicus (LSC), independent of atopic dermatitis (AD). We also evaluate advances in biologics and small-molecule therapeutics, exploring how targeted immune modulation may restore microbial balance and alleviate neuroinflammation. A systems biology approach integrating microbial genomics, neurobiology, and host immunity is critical to unraveling the complex interplay between host and microbes in chronic itch, particularly in understudied non-atopic conditions that disproportionately affect vulnerable populations.

## 1. Introduction

Acute itch is the unpleasant sensation leading to scratching behavior and is typically a physiologic response. Chronic itch is defined as pruritus lasting greater than six weeks and is associated with decreased quality of life, comorbid mood disorders, loss of sleep, and decreased daytime alertness [1,2,3]. Female sex, multiracial ethnicity, and stress are strong predictors of poor quality of life in those with chronic itch, and the relationship between itch severity of self-reported itch, sleep, and work productivity is strongly associated [4,5]. Chronic itch also disproportionately affects Black and Latino populations, with factors contributing to the racial and ethnic disparity largely being unknown [6,7,8,9,10]. A mechanistic understanding of itch has progressed significantly due to the large number of studies on atopic dermatitis (AD), a pruritic condition characterized by Th2-skewed immunity, skin microbiome dysbiosis, and atopy [11,12,13,14]. Non-atopic itch is less understood and includes disorders such as prurigo nodularis (PN) and lichen simplex chronicus (LSC). Recently, the FDA approved the first treatment for PN, although options remain limited [15].

The skin barrier, in conjunction with the skin microbiome, creates the initial defense against exogenous insults. While the link between skin microbiome dysbiosis and cutaneous disease pathogenesis in AD has been well-established, the contribution of the skin microbiome in non-atopic chronic itch conditions is less understood [13,16,17,18,19]. However, recent microbiome profiling studies are offering transformative insights into the role of dysbiosis in chronic itch [20,21]. We will review contributors to the itch–scratch cycle with a particular focus on components susceptible to modulation by the skin microbiome. Additionally, we highlight existing data on the skin microbiome in non-atopic chronic itch and discuss treatments targeting itch pathways with direct implications to the skin microbiome.

## 2. Materials and Methods

### 2.1. Literature Search Strategy

This narrative review synthesizes the peer-reviewed literature on host–microbiome interactions relevant to chronic itch, with a focus on non-atopic conditions including PN and LSC. A comprehensive search was performed using PubMed, Scopus, and Web of Science databases. The detailed search strategy, including Medical Subject Headings (MeSH) terms, keywords, inclusion/exclusion criteria, filters applied, and number of ingal studies included, are summarized in Table 1.

### 2.2. Eligibility and Selection Criteria

Included studies met the following criteria: (1) peer-reviewed primary research or reviews with a focus on skin microbiota and chronic itch; (2) use of molecular, genomic, or immunological assays to investigate microbial–host interactions; (3) studies involving human participants or mammalian models. Exclusion criteria included studies with a sole focus on AD, insufficient microbiome characterization, or studies limited to acute itch. A total of 120 studies were included in this review, as detailed in Table 1.

### 2.3. Data Extraction and Synthesis

Data extracted from eligible studies included microbial composition (species/genus level), immune and neuronal signaling markers, disease context, study population characteristics, and therapeutic interventions. Where available, sequencing platform and analytic pipelines (e.g., 16S rRNA vs. metagenomics, QIIME2) were noted. Findings were synthesized into thematic categories including barrier dysfunction, neuroimmune signaling, protease activation, and therapeutic modulation. No formal meta-analysis was performed due to heterogeneity in study design and outcome reporting.

### 2.4. Ethics Statement

As this manuscript is a literature-based review and does not involve new research with human or animal subjects, ethical approval was not required.

### 2.5. Data and Material Availability

This review does not include original experimental data. All supporting literature cited is publicly available through academic databases or open-access sources. No proprietary datasets or materials were used. There are no restrictions on material or information availability associated with this publication.

## 3. Results

**Figure 1:**
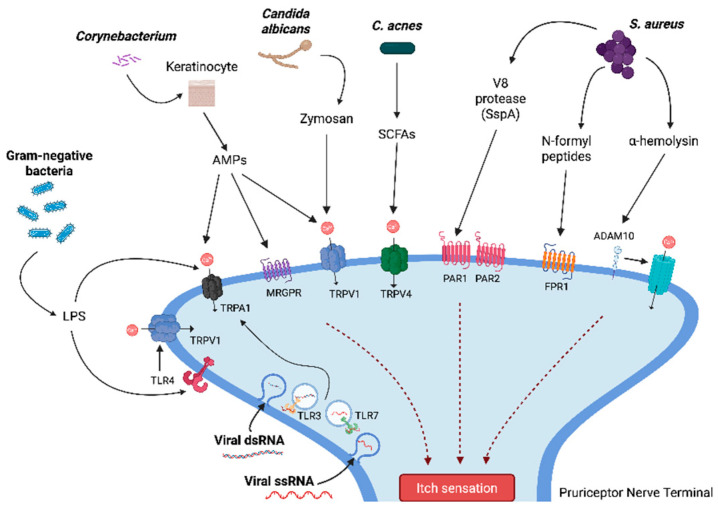
Microbial and viral ligands activate pruriceptive neurons through distinct ion channels and receptors, converging on itch sensation (Antimicrobial peptides (AMPs), short-chain fatty acids (SCFAs), transient receptor potential ankyrin 1 (TRPA1), transient receptor potential vanilloid 1 (TRPV1), transient receptor potential vanilloid 4 (TRPV4), Mas-related G-protein coupled receptor (MRGPR), protease-activated receptor 1 (PAR1), protease-activated receptor 2 (PAR2), formyl peptide receptor 1 (FPR1), Toll-like receptor 3 (TLR3), Toll-like receptor 7 (TLR7), lipopolysaccharide (LPS), double-stranded RNA (dsRNA), single-stranded RNA (ssRNA)).

This review synthesizes evidence highlighting the multifaceted contributions of the skin microbiome to chronic itch pathogenesis, particularly in non-atopic conditions. Key deregulated pathways include epidermal barrier disruption, neuroimmune signaling, cytokine-mediated inflammation, and microbial modulation of sensory neurons (Figure 1). The following subsections present findings from molecular, microbiological, and translational studies organized by mechanistic themes.

### 3.1. Microbial Dysbiosis in Non-Atopic Itch

Microbial dysbiosis in non-atopic itch involves an imbalance of the skin microbiome, with a reduction in beneficial commensal microbes like *Staphylococcus epidermidis* and an overgrowth of pathogens such as *Staphylococcus aureus* [22,23,24]. This imbalance disrupts the skin barrier through multiple mechanisms, enabling microbes and their byproducts to infiltrate deeper layers and trigger inflammatory cascades [25]. Pathogenic bacteria secrete proteases and toxins that compromise the skin barrier and stimulate protease-activated receptors (PAR-2) on sensory neurons, intensifying itch through direct neuronal activation [26].

Early bacterial studies in itch showed increases in *S. aureus*; however, these approaches were culture-based and were confounded by atopy and increased IgE [27,28]. Advances in microbiome analyses methods have allowed for a better understanding of microbial composition unattainable by traditional culture techniques. Kim et al. sampled two different populations in the United States and Korea with PN or LSC in the absence of other atopic conditions and performed 16S ribosomal RNA sequencing of chronic scratch lesions and healthy contralateral controls [20]. In chronic scratch lesions, *Staphylococcus* and *Cutibacterium* were the predominant genera, with the most abundant species being *S. aureus.* The copy number of the *S. aureus* protease, SplD, was increased in chronic itch lesions compared to healthy controls, suggesting a mechanistic link between bacterial virulence factors and pruritus.

Tutka et al. validated this study by comparing the skin microbiome of 34 patients with PN, 15 patients with AD, and 38 healthy subjects [21]. While alpha-diversity did not differ between groups, a significant difference was observed in beta-diversity. The genus *Staphylococcus* was significantly more abundant in both AD and PN groups compared to healthy controls, while no taxa differentiated PN from AD. This study addressed similarities between chronic itch conditions and atopic conditions; however, it was limited by the inability to differentiate bacterial species in the analysis. The current understanding of the PN-associated microbiome would further be improved with species–specific identification through metagenomic and meta transcriptomics approaches filling in the gap of knowledge on species and strain-level variation, in addition to functional bacterial gene expression profiles, for an in-depth understanding of itch-associated dysbiosis.

A comparative summary of microbiome characteristics in PN, LSC, and AD is provided in Table 2 to highlight shared and distinct microbial features across these chronic itch conditions.

### 3.2. Host–Microbiome Interaction in Chronic Itch: Molecular Pathways

#### 3.2.1. Epidermal Barrier Function and Microbial Interactions

Healthy skin is a functional and physical barrier preventing entry from a variety of exogenous factors, including microorganisms. Skin commensals contribute to barrier function by regulating epithelial differentiation, complementing the physical barrier formed by the stratum corneum, which is composed of dead keratinocytes and filaggrin (FLG) processing [35]. Degradation of FLG by caspase-14 leads to the production of natural moisturizing factor (NMF), and careful regulation is required to maintain barrier function, hydration, and pH [11,25]. Studies have shown that those with FLG variants are more likely to be predisposed to itchy conditions such as AD and have complete penetrance in Ichthyosis Vulgaris [36]. Epidermal barrier disruption itself is a potent activator of inflammation and itch, with many of the components of epidermal barrier integrity being weakened by the presence of pathogens, particularly *S. aureus*. Additionally, the act of repetitive scratching alone can also lead to a reduction in FLG, propagating the itch–scratch cycle [12]. In FLG-deficient mice, infection with *S. epidermidis*, a common skin commensal, shifts CD4+ T cell response from regulatory effector T-cell phenotypes, demonstrating how FLG variations shift adaptive immune responses to commensal organisms from tolerance to inflammation, while also activating itch pathways [37].

The skin’s physical barrier is strengthened by a lipid-rich matrix that maintains hydration and prevents water loss, known as lamellar membranes [38]. These membranes are derived from lamellar bodies, specialized secretory vesicles released by keratinocytes that deliver lipids and enzymes into the extracellular space of the stratum corneum. In addition to the maintenance of hydration, lipids are also an abundant source of nutrients for skin commensals, with numerous bacteria secreting molecules with surfactant properties to mobilize free fatty acids (FFAs) [38]. Commensal microorganisms incapable of synthesizing their own FFAs may also use synthesized FFAs released through other commensal species. The common commensal *S. epidermidis* secretes sphingomyelinase, aiding in the production of ceramides by keratinocytes and its own ability to acquire nutrients [39]. Keratinocyte treatment with *Cutibacterium acnes* conditioned media led to an increase in triglyceride accumulation and lipid synthesis upregulation [40]. On the contrary, a reduced lipid barrier leads to barrier dysfunction and an increase in trans epidermal water loss (TEWL), ultimately leading to dry and potentially itch prone skin. Elevated skin pH in dry skin states activates proteases leading to activation of PAR-2, feeding into the itch–scratch cycle [41]. Increased pH promotes the activity of serine proteases like kallikrein 5 (KLK5), which subsequently activate proteinase-activated receptor 2 (PAR2) on sensory neurons, triggering pruritus and inflammation [26,41]. Additionally, the activation of protease pathways fosters the secretion of thymic stromal lymphopoietin (TSLP), a cytokine that amplifies Th2-mediated inflammatory responses [41,42]. Recent findings highlight the role of *S. aureus* proteases, specifically the V8 protease (SspA), which directly activates PAR1 on sensory neurons, inducing significant itch and barrier damage. The interplay between PAR1 and PAR2 activation suggests a multifaceted role of these receptors in pruritus, mediated both by intrinsic skin proteases and microbial factors. Deng et al. demonstrated that inhibiting the SspA–PAR1 axis can mitigate *S. aureus*-induced itch and barrier damage, making this pathway a promising therapeutic target [26].

Although its relevance to chronic itch remains unclear, short-chain fatty acids from *C. acnes* have been shown to inhibit *S. epidermidis* biofilm formation, while other metabolites from common skin commensals can protect against pathogenic strains [43]. Lipids synthesized by the commensal species *Corynebacterium* exhibit antimicrobial properties and stimulate keratinocytes to produce antimicrobial peptides (AMPs) [44,45], crucial for chemical defense against exogenous insults and may also contribute to itch by promoting the release of β-defensins, endogenous pruritogens that directly activate sensory neurons. β-defensins are thought to play a significant role in the induction of itch through their interaction with sensory neurons [46]. Elevated expression of β-defensins, such as DEFB102, is observed in inflammatory skin conditions like psoriasis and AD, correlating with chronic itch symptoms [46]. These peptides act as endogenous pruritogens by directly stimulating sensory neurons via Mas-related G protein-coupled receptors (MRGPRs), specifically MRGPRa 3 in mice and MRGPRX1 in humans [46]. In mice, β-defensins have been shown to induce scratching behavior through Toll-Like Receptor 4 (TLR-4)-dependent and MRGPR-mediated mechanisms [46]. Interestingly, MRGPR2a knockout mice display reduced microbial diversity and overrepresentation of *Staphylococcus* species. This phenomenon was further exemplified by the inability to control an epicutaneous *S. aureus* infection in knockout mice [47]. TLR3, another Toll-like receptor, is critical in recognizing double-stranded RNA and initiating immune responses. Expressed on keratinocytes, immune cells, and pruriceptors, TLR3 activation by its ligand, PolyI:C, stimulates neuronal activity and promotes itch [25]. TLR3 activation also triggers the release of pro-inflammatory cytokines such as IL-6, TNF-α, and type I interferons, which sensitize sensory neurons [25]. Dysbiosis of the skin microbiome can amplify TLR3-driven inflammation by disrupting the skin barrier and exposing nucleic acid mimics. Additionally, TLR3 activation upregulates keratinocyte-derived TSLP, which amplifies Th2 inflammation and pruritic signaling. However, this TLR3-dependent inflammation can be adjusted by introducing TLR2-mediated crosstalk induced by Gram-positive commensal bacteria, which subdues inflammation, providing an additional regulatory mechanism.

#### 3.2.2. Itch-Related Cytokines as Pruritogens

As pruritogens breach the epidermal barrier, they encounter immune cells, triggering innate and adaptive immune responses. Aberrant, enhanced type 2 inflammation, characterized by overexpression of IL-4, IL-13, and IL-31, is known to be characteristic of disorders in which itch is a predominant concern. Th2 cytokine receptors are expressed on human DRG neurons and can be activated by Th2 cytokines, making them direct players in itch signaling [48]. IL-4, produced by Th2 cells, mast cells and eosinophils, drives B cell proliferation and IgE isotype switching. IL-13, another potent inducer of the Th2 response, shares overlapping functions with IL-4. These cytokines contribute to chronic itch by directly binding to sensory DRG, increasing sensitivity to other pruritogens and reducing FLG expression [49,50]. IL-4 and IL-13 overexpression has been observed in AD and PN; however, their impact on the skin microbiome remains less understood [51]. In mice, IL-4 deficiency leads to decreased AMPs and inhibition of coagulase-negative *Staphylococcal* species (CoNS), resulting in *S. aureus* overgrowth [52]. In humans, IL-4/IL-13 blockade has led to remarkable insight into the systemic effects of Th2 inflammation on the skin barrier integrity and skin microbiome homeostasis [33]. Particularly, Dupilumab, a human monoclonal IL-4ra/IL-13 antibody, and Lebrikizumab, an IL-13-specific monoclonal antibody [53,54].

Dupilumab has recently become approved for use in PN given its efficacy in reducing itch and PN skin lesions when compared to placebo [15,55,56,57]. Several studies in AD have shown a reduction in *S. aureus* colonization within three days of Dupilumab treatment, accompanied by increased microbial diversity [58,59]. These microbiome shifts precede visible clinical improvement by several days and are accompanied by decreased *S. aureus* cytotoxin production through an IL-17-dependent mechanism in both lesional and non-lesional skin [59]. Similar dysbiosis-correcting effects are being investigated with Nemolizumab, an IL-31 receptor-α antagonist, which has shown early promise in re-normalizing the skin microbiome in AD and PN cohorts [60]. Together, longitudinal strain-level profiling before and after biologic therapy provides a powerful lens through which to dissect the neuro–immune–microbial circuitry driving chronic itch.

IL-33, a member of the IL-1 family, amplifies the Th2 immune response and contributes to chronic itch. *S. aureus* uniquely induces IL-33 release from keratinocytes, disrupting barrier function and potentially contributing to non-atopic itch conditions [61]. Despite its importance, bacterial-induced keratinocyte IL-33 secretion remains underexplored.

The Janus kinase (JAK)/signal transducer and activation of transcription (STAT) pathway has been the target of a new class of therapeutics that are increasingly utilized in dermatology, including the treatment of itch. Abrocitinib, an oral Jak1 inhibitor, offers rapid itch relief, with significant improvement observed within days [62]. While effective, the impact of these treatments on the skin microbiome remains unknown, warranting further investigation into how inflammatory pathways influence microbial composition.

Given the interplay between immune signaling and microbial composition, modulation of Type 2 inflammation may have a potential to restore microbial balance, further alleviating itch and enhancing skin barrier function. Understanding this dual effect, on both inflammation and the skin microbiome, could pave the way for more comprehensive treatment strategies, emphasizing the need for further research to optimize therapeutic outcomes.

#### 3.2.3. Protease-Activated Receptors and Microbial Dysbiosis

Protease-activated receptors (PARs) are transmembrane G-protein-coupled receptors activated by protease cleavage [63]. They are ubiquitous and play a role in various physiologic processes, including coagulation and cytokine release. PAR-2, expressed in keratinocytes, has been extensively studied in AD and is associated with barrier disruption, tight junction disruption, inflammation, and pruritus [64,65]. Notably, Protease-activated receptor 1 (PAR1) activation can also be triggered by secreted microbial products, multiple *S. aureus* proteases [26]. *S. aureus* secretes serine protease-like proteins (Spls), encoded by the spl operon and including SplA-SplF [66]. These proteases are assumed to enhance *S. aureus* virulence, as the psl operon is flanked by other virulence factors such as enterotoxins and leukocidins. In our work, we found that the copy number of the *S. aureus* protease *SplD* is increased in chronic scratch lesions when compared to healthy controls [67]. Proteases in the stratum corneum, especially those secreted by *S. aureus*, activate keratinocytes and neurons, driving inflammation and perpetuating the itch–scratch cycle. Therefore, it is crucial to study these proteases in a host of chronic itch disorders independent of atopy to determine pathways induced or exacerbated by the presence of pathogens and microbiome dysbiosis and to find unique targets for therapeutics in non-atopic itch [20].

Lastly, keratinocytes can secrete their own proteases in response to insult, a crucial step in maintaining skin homeostasis [68]. For example, Kallikreins (KLKs) are serine proteases with trypsin- or chymotrypsin-like activity. Excessive expression of KLKs is observed in AD and is believed to lead to allergen and pathogen penetration [69]. *S. aureus* further exacerbates this process by inducing KLK expression in keratinocytes, as demonstrated in mouse models [69,70]. Specific KLKs, such as KLK6, KLK13, and KLK14, are associated with itch by activating PAR2s on sensory nerves, which transmit itch signals. KLKs overexpression weakens the skin barrier by degrading corneodesmosomes, facilitating allergens and pathogens entry, triggering inflammatory responses, and further sensitizing itch receptors [71]. Taken together, these studies suggest that pathogens can initiate and propagate vicious cycles of protease activity that can potentially interact with PARs. It is unclear if other cutaneous pathogens can secrete PAR-activating proteases or if there is a regulatory or inhibitory effect of skin commensals.

#### 3.2.4. Transient Receptor Potential Channels and Microbiome

Pruritogens penetrate the epidermal barrier, and, in combination with inflammatory mediators, receptor activation takes place at the site of the sensory neuron. Transient receptor potential channels (TRPs) are cation-permeable channels that, when opened, lead to depolarization and, ultimately, the sensation of itch. Transient receptor potential channel 1 (TRPV1) is activated by histamine, capsaicin, and lipoxygenase products and plays a role in both histaminergic and non-histaminergic pathways [72]. TRPV1+ neurons are sensory afferent neurons whose endings are dispersed throughout the skin, capable of detecting pain and itch. These neurons also interact with key players in cutaneous immunity, such as dendritic cells, and serve a pivotal function in mediating Th17 immune responses [73]. TRPV 1 and TRPA1 are ion channels on sensory afferents that can be activated by *S. aureus* to evoke itch, whereas TRPV, a thermosensitive channel highly expressed in keratinocytes and upregulated when the epidermal barrier is damaged, has not been definitively implicated in *S. aureus*-driven pruritus [72]; beyond their pruritogenic roles, neurons bearing TRPV1 and TRPV3 also participate prominently in pain signaling [7,72].

The skin microbial ecosystem influences TRP channel activity and its downstream effects (Figure 1). TRP channels not only mediate sensory responses, such as pain and itch, but also play a critical role in immune modulation by interacting with dendritic cells to shape cytokine pathways, including IL-17-driven responses. This intricate relationship underscores the dual function of TRP channels as regulators of sensory perception and immune homeostasis, highlighting their potential as therapeutic targets in managing infection and inflammation.

Dysbiosis significantly impacts TRP channel regulation, fueling chronic inflammation, pain, and pruritus. Overgrowth of pathogenic bacteria such as *S. aureus* introduces microbial toxins, including a-Hla, which directly activate TRPV1+ neurons by forming transmembrane pores, leading to heightened pain sensitivity and inflammation (Figure 1) [74]. Additionally, *S. aureus* releases proteases and other virulence factors that sensitize TRP channels, promoting the release of neuropeptides like CGRP and Substance P [75,76] (Table 3). These neuropeptides amplify local immune responses while also impairing neutrophil recruitment, creating a microenvironment favorable for bacterial survival. Additionally, the loss of beneficial microbial metabolites, including short-chain fatty acids produced by *C. acnes*, further dysregulates TRP channel activity, impairing skin barrier function and exacerbating inflammatory responses [40,73]. Loss of these protective effects exacerbates TRP dysregulation, inflammatory responses, and chronic skin conditions [72]. This reciprocal relationship between dysbiosis and TRP activity perpetuates sensory and immune dysfunction in inflammatory skin diseases.

#### 3.2.5. Neuropeptides

Neuropeptides are crucial mediators of itch and pain transmission from the peripheral nervous system to the central nervous system, involving molecules like Substance P, CGRP, brain natriuretic peptide (BNP), acetylcholine, histamine, and catecholamines mediating in complex neuroimmune interactions [75,76,80,81]. Recent studies have highlighted the intricate crosstalk between the skin microbiome and neuropeptides, demonstrating how microbial products and host neuropeptides collaboratively shape neuronal activity and skin immune responses [82,83].

Substance P, a primary neuropeptide released by C-fiber sensory neurons, has been implicated in skin conditions such as AD, PN, and chronic urticaria [84,85]. Within the epidermis, Substance P promotes antigen presentation, allergic sensitization, and histamine release from mast cells [77,86]. Importantly, it also enhances the virulence of *Staphylococci* in reconstructed skin models by increasing adhesion and enterotoxin production, suggesting its role in fostering microbial dysbiosis in pruritic states [73]. Co-secreted with CGRP, Substance P amplifies neurogenic inflammation and immunocyte activation. CGRP, released upon TRPV1 activation, induces vasodilation and cytokine production, such as IL-4 and IL-13 [73]. Furthermore, CGRP has been shown to enhance the *S. epidermidis* cytotoxicity, modulate keratinocyte adherence, and stimulate robust antimicrobial peptide responses through a DnaK-dependent mechanism [87]. These neuropeptide-driven alterations in host–microbe interactions may contribute to shifts in microbial community structure and increased susceptibility to skin inflammation.

Beyond *Staphylococci*, species like *Corynebacterium* also contribute significantly to neuropeptide-mediated signaling. *Corynebacterium*-derived bioactive lipids and metabolites interact with sensory neurons and keratinocytes to regulate the release of Substance P, CGRP, and vasoactive intestinal peptide (VIP) [88]. These microbial signals influence host receptor activation, such as TLR pathways, enhancing antimicrobial peptide production in response to neuropeptides. *Corynebacterium* also modulates sensory neuron activation thresholds, balancing excitatory and inhibitory signaling to fine-tune neurogenic inflammatory responses. Dysbiosis or reduced functionality of *Corynebacterium* has been linked to heightened neuropeptide activity, exacerbating the chronic inflammation and itch associated with AD and PN [89].

Moreover, BNP, a central itch mediator, exemplifies the bidirectional influence between neuropeptides and microbes. Upregulated by IL-31 in dorsal root ganglia, BNP is detectable by *P. aeruginosa* through a BNP receptor ortholog [90]. BNP exposure enhances *P. aeruginosa* virulence, while its bacterial protein Atrial Natriuretic Peptide-Modulating Cytotoxicity Inhibitor mimics BNP binding to host receptors, reducing biofilm formation and promoting a more virulent state [91]. Although *P. aeruginosa* is not classically implicated in itch, these findings suggest that neuropeptide-driven changes in microbial behavior, particularly enhanced virulence and host interaction, may contribute to inflammatory cascades that indirectly promote pruritus in compromised skin environments. Together, these findings highlight the intricate and bidirectional communication between the skin microbiome and neuropeptides, with significant implications for skin inflammation and itch pathophysiology.

#### 3.2.6. Microbial Metabolites and Sensory–Neuroimmune Crosstalk

Emerging research highlights the critical role of microbial metabolites in modulating neuroimmune pathways implicated in both atopic and non-atopic chronic itch. One such metabolite, indole-3-aldehyde (IAld), is significantly diminished in AD skin and attenuates inflammation via aryl hydrocarbon (AhR) activation and subsequent suppression of TSLP [92]. In AD, non-histaminergic itch is driven by microbial factors—including bacterial proteases, elevated skin pH, and neuropeptide–microbiome interactions—that activate peripheral sensory neurons through PARs, TLRs, and MRGPRs [71,76,83,93]. In non-atopic conditions such as PN and LSC, microbial-derived small molecules similarly mediate neuroimmune crosstalk, driving itch pathogenesis through both direct neuronal activation and immune modulation [94]. For instance, *S. aureus*-secreted proteases, including SspA, activate PAR1 on sensory neurons, inducing pruritic signaling via inflammatory and neuronal pathways [26]. Lipopolysaccharides from Gram-negative bacteria sensitize sensory neurons indirectly through TLR4 activation, amplifying downstream cytokine release [26,78,95]. Commensal-derived SCFAs support immune tolerance by enhancing regulatory T cell (Treg) function, while dysbiosis-associated reductions in SCFAs promote inflammation and neuronal hypersensitivity ([96,97]). Similarly, tryptophan metabolites produced by skin commensals activate AhR signaling, regulating keratinocyte function and modulating pruritogen production [98]. Together, these findings emphasize the role of microbial metabolites in bridging microbial ecology with neuroimmune signaling in chronic itch.

Recent research in a murine allergic-contact-dermatitis model shows that mechanical scratching is indispensable for both the ensuing inflammation and for limiting superficial *Staphylococcus aureus* infection [99]. Scratching activates TRPV1^+^ nociceptors, triggering substance P release that engages the mast-cell receptor MrgprB2 and synergizes with classical FcεRI signaling, thereby amplifying TNF-driven neutrophil recruitment and mast-cell degranulation. Mice prevented from scratching or lacking NP2 itch-sensing neurons fail to achieve this antibacterial effect, underscoring the protective dimension of the itch–scratch reflex [99]. Although these findings derive from an acute model, they suggest that mechanical cues can transiently reshape cutaneous microbial communities and may help explain the rapid reductions in *S. aureus* detected after anti-itch biologics, providing a framework for probing neuro–immune–microbial interactions in chronic pruritic disorders.

While bacterial contributors such as *S. aureus, S. epidermidis,* and *C. acnes* dominate current chronic itch research, non-bacterial microbes may also influence disease. Fungal species, particularly *Malassezia*, have been implicated in the exacerbation of skin inflammation through their metabolic byproducts and interactions with keratinocytes and immune cells [100,101]. Dysbiosis involving fungal overgrowth may contribute to barrier disruption and cytokine-mediated pruritus, though data specific to PN and LSC remain limited [21,79].

Viral pathogens represent another underexplored dimension of the chronic itch microbiome. Herpesviruses, through periodic reactivation and latent infection, can trigger sustained immune dysregulation in inflammatory dermatoses, potentially contributing to the chronicity of pruritic conditions through mechanisms that extend beyond the acute infection phases [102,103,104]. However, the direct contribution of viral components to itch sensation and the interplay between viral, bacterial, and fungal communities in chronic pruritic states remain poorly characterized. Comprehensive multi-kingdom microbial profiling, incorporating mycobiome and virome analyses alongside traditional bacterial approaches, will be essential to fully illuminate host–microbiome interactions driving non-atopic chronic itch pathogenesis.

#### 3.2.7. Convergent Pathways and Feedback Loops in Chronic Itch

The molecular mechanisms underlying chronic itch function as an interconnected network rather than discrete pathways. Rather than linear activation cascades, these pathways demonstrate extensive bidirectional crosstalk that amplifies initial stimuli and creates persistent pathological states resistant to single-target interventions [50].

Elevated skin pH and barrier disruption activate serine proteases (e.g., KLK5), which stimulate PAR2 on keratinocytes and sensory neurons, initiating itch and inflammation [41]. This environment facilitates colonization by *S. aureus*, whose virulence factors, including V8 protease, directly trigger neuronal activation via PAR1 [22,26]. Simultaneously, both microbial and host-derived proteases influence TRP channel sensitivity through downstream calcium signaling, with PAR2 activation enhancing TRPV1 responsiveness to subsequent stimuli [105,106].

Neuropeptides like Substance P and CGRP, released from activated sensory fibers, drive mast cell degranulation and neurogenic inflammation while directly modulating microbial behavior [84,85]. Substance P enhances *S. aureus* virulence and adhesion, creating feed-forward loops where neuronal activation promotes conditions for further microbial stimulation [80].

The relative contributions of these pathways shift temporally, with direct neuronal activation (PARs, TRP channels) predominating in acute responses while chronic states become dominated by cytokine-mediated sensitization and barrier dysfunction feedback loops [107,108]. This temporal evolution explains why treatments effective in acute settings may have limited efficacy in established chronic conditions. These mutually reinforcing mechanisms explain why multi-pathway targeting approaches like Dupilumab demonstrate superior efficacy compared to single-pathway inhibition [109]. Dupilumab’s clinical success stems from its simultaneous disruption of cytokine signaling, barrier restoration, and microbiome normalization, necessitating multi-modal therapeutic strategies that target complementary pathway nodes to achieve synergistic effects in chronic itch management [109,110].

## 4. Discussion

Itch is a complex entity with the mechanisms of chronic itch remaining elusive and understudied. We have outlined the many contributors to the itch–scratch cycle and the known interactions with bacterial pathogens and commensals, highlighting the importance of the skin microbiome in exacerbating and preventing itch (Table 3). While many studies focus on AD, limited data exists in non-atopic itch. We and others have documented skin microbiome dysbiosis in chronic itch conditions, but there is a growing need to combine genomic, meta-transcriptomic and model-based approaches to study species-specific effects on the itch–scratch cycle. In addition to microbial and host factors, real-world environmental exposures, such as hygiene practices, harsh soaps, cosmetic use, and topical products, may also contribute to microbial dysbiosis in chronic itch. In conditions like PN and LSC, these exposures can disrupt the lipid barrier and deplete commensal microbes, creating a permissive environment for *S. aureus* colonization [23,31,38]. This shift in microbial composition may further impair barrier function and promote immune activation and neuronal sensitization, ultimately perpetuating the itch–scratch cycle and contributing to disease persistence [38]. As such, future studies examining host–microbiome interactions in chronic itch should consider these environmental variables as potential modulators of microbial signatures, symptom severity, and treatment response.

Importantly, with the availability of advanced biologic and small-molecule therapies, there is an opportunity to study the effects of immune modulation on the skin microbiome to provide insight into possible mechanisms of neuroinflammation. Further translational and/or clinical studies focused on the skin microbiome in chronic itch will need to focus on multiple anatomical sites, include validated patient-reported outcome measurements, and incorporate various biomarkers of itch, including those listed above. Longitudinal analysis of microbiome–immune dynamics in response to treatment could offer key mechanistic insights. Interventional trials evaluating how targeted microbial restoration or immune modulation (e.g., topical probiotics, IL-31 inhibitors) impact pruritus severity and microbiome composition would provide mechanistic and clinical insight. As chronic itch has significant morbidity and mortality in patients, especially those with skin of color, it is of the utmost importance that future studies include racially and ethnically diverse populations to differentiate social, environmental, and biologic contributions to disease pathogenesis.

## Figures and Tables

**Table 1:** Summary of Literature Search Strategy for Narrative Review on Host–Microbiome Interactions in Chronic Itch.

MeSH Terms	Pruritus; Skin Microbiome; Dysbiosis; Neuroimmunomodulation; TRPV Cation Channels; Interleukin-31; Filaggrin; Host–Pathogen Interactions; Chronic Skin Diseases; Non-Atopic Dermatitis; Signal Transduction; T-Lymphocytes
Keywords	Chronic itch; prurigo nodularis; lichen simplex chronicus; skin dysbiosis; microbiota imbalance; neuroimmune signaling; protease-activated receptors; TRP channels; IL-31; filaggrin mutation; biologics AND pruritus; immune-microbiome crosstalk; skin barrier dysfunction; inflammatory mediators; cutaneous nerves; Staphylococcus aureus; cytokine signaling; epithelial-immune interactions
Databases Searched	PubMed, Scopus, Web of Science
Date Range	January 1991–June 2025
Language and Filters	English language only; humans (where applicable); article types limited to primary research articles, systematic reviews, and narrative reviews
Inclusion Criteria	Peer-reviewed primary studies or reviews. Focus on skin microbiota and chronic (non-atopic) itch. Use of molecular, genomic, or immunological assays to study host–microbiome interactions. Studies in human participants or mammalian models.
Exclusion Criteria	Studies with insufficient microbiome characterization. Articles addressing only acute or transient itch.
Additional Sources	Reference lists of eligible articles and relevant reviews were manually screened.
Final Studies Included in Review	111

**Table 2:** Comparative microbiome characteristics in chronic itch conditions.

	Atopic Dermatitis (AD)	Lichen Simplex Chronicus (LSC)	Prurigo Nodularis (PN)
Dominant Pathogen(s)	*Staphylococcus aureus* [29,30]	No dominant pathogen	*Staphylococcus aureus* [21,31]
Commensal Depletion	*Cutibacterium, Streptococcus, Acinetobacter, Corynebacterium, and Prevotella* [29,32,33]	Not well characterized	Marked increase in the abundance of *Staphylococcus aureus* in lesional skin [21,31,34]
Microbial Diversity	Reduced alpha diversity [29,32,33]	Not well characterized	Reduced alpha diversity at lesional sites [21,31,34]

**Table 3:** Microbial contributions to chronic itch: mechanisms and potential therapeutic strategies.

**Bacterial Species**	**Proposed Mechanism of Itch**	**Potential Therapeutic Strategies**
*Staphylococcus aureus*	Releases proteases (e.g., SplD, V8/SspA) activating PAR1/2 [24,25,36,76]; α-hemolysin forms pores via ADAM10 [77]; induces IL-31 and IL-33 [63,70]	IL-4/IL-13 blockade (e.g., Dupilumab); IL-31RA antagonism (e.g., Nemolizumab); PAR1 inhibitors; ADAM10 inhibition [19,36,64]
*Cutibacterium acnes*	Produces short-chain fatty acids (SCFAs) that regulate TRPV4 and enhance lipid barrier function [34,38]	Lipid barrier repair; probiotic-derived SCFA supplementation; keratinocyte lipid modulation [34,38]
*Corynebacterium* spp.	Secretes bioactive lipids that stimulate AMP release and interact with neuropeptides (Substance P, CGRP) [39,40,78]	Topical AMP inducers; bioactive lipid therapy; support commensal colonization [39,40]
*Staphylococcus epidermidis*	Promotes ceramide synthesis via sphingomyelinase; secretes AMPs; suppresses S. aureus biofilm [33,38]	Enhance barrier commensals; prebiotic support for ceramide pathways [33,38]
*Gram-negative bacteria* (e.g., *Pseudomonas* spp.)	Lipopolysaccharide (LPS) activates neuronal TLR4 → sensitizes TRPV1 and activates TRPA1, inducing itch [21,79]	TLR4 or TRPA1 antagonists; anti-inflammatory biologics to reduce cytokine-mediated sensitization [21,79]

## Data Availability

No new data were created or analyzed in this study. Data sharing is not applicable to this article.

## Abbreviations

The following abbreviations are used in this manuscript:

AD	Atopic dermatitis
AMCi	Atrial Natriuretic Peptide-Modulating Cytotoxicity Inhibitor
AMP	Antimicrobial peptide
BNP	Brain natriuretic peptide
CGRP	Calcitonin gene-related peptide
CKD	Chronic kidney disease
CoNS	Coagulase-negative staphylococci
CTCL	Cutaneous T-cell lymphoma
DRG	Dorsal root ganglia
FDA	U.S. Food and Drug Administration
FFA	Free fatty acid
FLG	Filaggrin
IL	Interleukin
JAK	Janus kinase
KLK	Kallikrein
LSC	Lichen simplex chronicus
MRGPR	Mas-related G protein-coupled receptor
NMF	Natural moisturizing factor
PAR	Protease-activated receptor
PN	Prurigo nodularis
SCFA	Short chain fatty acid
SIR	Standardized incidence ratio
STAT	Signal transducer and activator of transcription
TEWL	Transepidermal water loss
TLR	Toll-like receptor
TRP	Transient receptor potential
TRPV1/3/4	Transient receptor potential vanilloid channels 1, 3, and 4
TSLP	Thymic stromal lymphopoietin
VIP	Vasoactive intestinal peptide
